# Analysis of comorbid characteristics, molecular mechanisms between mild cognitive impairment and Alzheimer’s disease with interventions in a community-based population in Shanghai

**DOI:** 10.3389/fneur.2026.1765285

**Published:** 2026-01-29

**Authors:** Yiying Sun, Bin Liu, Jie Tong, Dianhong Shi, Xiaochun Zhu, Tingting Jiang, Yi Yang, Xirong Sun

**Affiliations:** Shanghai Pudong New Area Mental Health Center (Tongji University Affiliated Mental Health Center), Tongji University Clinical Research Center for Mental Disorders, Shanghai, China

**Keywords:** AD, comorbid characteristics, intervention community, MCI, Shanghai

## Abstract

**Background:**

Mild cognitive impairment (MCI) is a critical early stage of Alzheimer’s disease (AD). Elucidating the comorbidity characteristics, influencing factors, and molecular mechanisms between MCI and AD in community-based populations is crucial for early intervention in cognitive impairment.

**Methods:**

2,234 elderly individuals aged 50 years or older from 14 communities in Pudong New District, Shanghai, were enrolled in this study. The Montreal Cognitive Assessment (MoCA), Mini-Mental State Examination (MMSE), and Clinical Dementia Rating (CDR) were used to divide individuals into a control group (*n* = 1,160) and an MCI group (*n* = 1,074). The associations of demographic characteristics, lifestyle, and psychological status with MCI were analyzed. Transcriptome data from GSE140829 (training set) and GSE63060 (validation set) were obtained from the GEO database. Weighted gene co-expression network analysis (WGCNA) was used to identify MCI signature genes. KEGG pathway analysis was combined with the Kyoto Encyclopedia of Genes and Genomes (KEGG) pathway to elucidate mechanisms of comorbidity. Targeted intervention agents were screened based on the DSigDB database. Molecular docking (MD) was used to evaluate the binding ability between small molecules and target proteins.

**Results:**

The prevalence of AD in the MCI group (30.17%) was significantly higher than that in the control group (*p* < 0.001), and MCI and AD were significantly positively correlated. Age, gender, smoking, living arrangements, mobile phone use, pet ownership, stress, anxiety, and depression were key influencing factors for MCI (*p* < 0.05). The proportion of individuals living with children and grandchildren (57.45%) in the MCI group was significantly higher than that in the control group (16.29%) (*p* < 0.001). WGCNA identified 273 MCI signature genes. KEGG pathway analysis showed that these genes were significantly enriched in neurodegenerative disease pathways, including AD pathways (with the AD pathway ranking first in the “Human Diseases” category). Targeted intervention screening identified the natural compounds boldine (comprehensive score 961.58) and piceatannol (comprehensive score 358.46) as potential drug candidates (*p* < 0.05), both of which have strong binding ability to target proteins.

**Conclusion:**

MCI patients in the community are at high risk of AD, and their comorbidity characteristics are affected by multidimensional lifestyle and psychological factors. Boldine and piceatannol may be potential natural compounds for the intervention of cognitive impairment. The results of this study can provide a theoretical basis for the early prevention and precise intervention of cognitive impairment in the community.

## 1 Introduction

The accelerated aging of the global population has made cognitive impairment-related diseases a major challenge in the field of public health. Mild cognitive impairment (MCI), a critical early stage of Alzheimer’s disease (AD) (1–3), has significant heterogeneity in its clinical outcomes. Approximately 10–15% of MCI patients may progress to AD, while some patients can maintain stable or even recover to normal cognitive function (4). This heterogeneity not only increases the difficulty of early clinical intervention, but also highlights the importance of understanding the comorbidity characteristics and potential mechanisms of MCI and AD. Existing studies exploring the association between MCI and AD have mostly focused on hospital patients. Such samples were often patients with severe conditions or multi-system diseases, which makes it difficult to reflect the true distribution and influencing factors of cognitive impairment in the natural community population (5). As a long-term living environment for the elderly, the community has a closer relationship between population characteristics and cognitive function. Community environmental factors, such as living alone and social isolation, have been proven to be associated with an increased risk of cognitive decline (6). However, there is still a lack of systematic analysis of the comorbidity patterns of MCI and AD in the community population, especially the lack of integrated research combining macro-epidemiological characteristics with micro-molecular mechanisms.

Transcriptomics provides an important tool for analyzing the molecular mechanisms of cognitive impairment. Dynamic changes in gene expression are the core molecular basis for the occurrence and development of the disease. Through methods such as differentially expressed genes (DEGs) screening and weighted gene co-expression network analysis (WGCNA), key gene modules and signaling pathways related to MCI and AD can be identified (7, 8). Previous studies have found that AD patients have abnormal synaptic function, neuroinflammation, and other pathway disorders in the brain (9), but these findings are mostly based on autopsy brain tissue or cell models, which are difficult to directly associate with the clinical phenotypes of community populations (such as lifestyle, psychological state, etc.), resulting in a disconnect between molecular mechanism research and clinical translation. In addition, current intervention studies for MCI still face the dilemma of “ambiguous targets,” and most candidate drugs failing due to a lack of targeting of specific molecular pathways for MCI and AD comorbidity (10). Screening targeted intervention agents related to comorbidity based on transcriptomics data can enhance the accuracy of interventions. Natural compounds have demonstrated potential in the intervention of cognitive impairment due to their advantages of low toxicity and high biocompatibility, but their applicability needs to be further verified in combination with the epidemiological characteristics of community populations (11).

This study used the community population in Pudong New Area, Shanghai, as the sample source. By integrating epidemiological surveys and transcriptomic data, the following aims were to: 1. clarify the epidemiological characteristics and key influencing factors of MCI and AD comorbidity in the community population; 2. screen for characteristic genes and signaling pathways associated with MCI-AD comorbidity; 3. identify potential natural compound targeted intervention agents to provide a theoretical basis for the early prevention and precise intervention of cognitive impairment.

## 2 Methods

### 2.1 Survey subjects

From November 2024 to October 2025, elderly people aged 50 and above were recruited as survey respondents from 14 communities in Pudong New Area, Shanghai. Exclusion criteria included: 1. patients with malignant tumors, severe liver and kidney dysfunction, or other serious complications; 2. currently hospitalized or residing in a nursing home; 3. patients with a history of alcohol dependence or psychoactive drug abuse; 4. patients with neurological disorders (such as stroke, brain tumors, and Parkinson’s disease).

### 2.2 Study variables and sample size calculation

The variables examined in this study included age, gender, smoking, alcohol consumption, income level, education level, marital status, living arrangements, mobile phone use, pet ownership, stress, anxiety, depression, and AD. Based on the rule of thumb of 10 events per variable (10 EPV) (12), the number of independent variables included in this study was 14, and the minimum sample size of 140 patients. This study was reviewed and approved by the ethics committees of the Shanghai Pudong New Area Mental Health Center and the Tongji University Mental Health Center.

### 2.3 Cognitive function assessment

The Montreal Cognitive Assessment (MoCA) and Mini-Mental State Examination (MMSE) were used. The assessment was completed by uniformly trained medical staff in a quiet clinic in the form of a one-on-one interview. Before the assessment, the participants were ensured to be conscious and free from interference. Special circumstances (visual/hearing impairment) were noted.

#### 2.3.1 Montreal cognitive assessment

The MoCA (13) covers seven dimensions, including attention, executive function, memory, and language, with a total score of 30 points. For those with ≤ 12 years of education, a score of ≥ 26 points is considered normal, and a score of < 26 points indicates cognitive impairment; for those with > 12 years of education, a score of ≥ 27 points is considered normal, and a score of < 27 points indicates cognitive impairment, with an emphasis on identifying mild cognitive impairment (MCI). If the number of years of education is less than 12 years, 1 point is added to the result to correct for educational bias.

#### 2.3.2 Mini-mental state examination

MMSE (14) assesses five dimensions, including orientation, memory, and attention, with a total score of 30 points. According to the educational level, the scores of illiterate people ≥ 17 points, primary school students ≥ 20 points, and middle school and above ≥ 24 points are considered normal. Scores below the corresponding values indicate cognitive impairment and are used for the rapid screening of cognitive abnormalities.

### 2.4 Dementia assessment

For individuals with cognitive impairment, the Clinical Dementia Rating (CDR) was used. Through cross-validation of “research subject interviews + informed supplements,” two qualified researchers jointly evaluated the scores. Disagreements are resolved through discussion or guidance from senior physicians. The CDR includes six dimensions, including memory (core), orientation, and daily living ability. The scores are scored from 0 to 3 points according to the degree of impairment. The final comprehensive judgment is: CDR = 0 points is normal, 0.5 points is suspected dementia, and ≥ 1 points is judged as dementia (1 point is mild, 2 points are moderate, and 3 points are severe).

### 2.5 Data acquisition and processing

Transcriptional expression profile data were obtained from the Gene Expression Omnibus (GEO) database^1^. Dataset GSE140829 was used as the training cohort, and dataset GSE63060 was used as the validation cohort. GSE140829 included 249 healthy controls and 133 patients with MCI, while dataset GSE63060 included 102 healthy controls and 78 patients with MCI. For preprocessing, each dataset underwent a mapping process, in which probes were aligned to their corresponding gene identifiers, and probes lacking significant signal were excluded. If multiple probes corresponded to a single gene, the median value was used as the representative value for that gene. Subsequently, the datasets were normalized, and differentially expressed genes were screened using the “limma” R package. Genes with a *p* < 0.05 and a fold change (FC) ≥ 2 were identified as DEGs.

### 2.6 Weighted gene co-expression network analysis (WGCNA)

The ‘WGCNA’ R (15) package was used to construct a WGCNA network for the GSE140829 and GSE63060 datasets to identify gene modules associated with MCI. We selected the top 50% of genes exhibiting the greatest variation as input for the analysis, determined the optimal soft threshold based on the scale-free topological structure criterion, and then constructed a weighted adjacency matrix and a topological overlap matrix. Hierarchical clustering was performed using a similarity threshold of 0.85, and modules containing more than 20 genes were identified and marked with unique colors. Further optimization analysis focused on modules that were significantly associated with the phenotype.

### 2.7 Functional enrichment analysis and GeneMANIA analysis

Cluster analysis package (16) and the pathview package (17) were used for Gene Ontology (GO) analysis and Kyoto Encyclopedia of Genes and Genomes (KEGG) pathway analysis. Gene Ontology functional enrichment analysis includes molecular functional analysis [MF], biological process analysis [BP], and cellular component analysis [CC]. The analysis of KEGG was used to identify important pathways for gene enrichment. GeneMANIA^2^ is a web-based tool that provides protein and genetic interaction information (18). By using GeneMANIA, interactions at the gene and protein expression levels were identified.

### 2.8 Targeted intervention screening

The DSigDB database includes 17,398 drugs and 19,531 drug-related genes, used for screening small molecule targeted drugs (19). The higher the comprehensive score, the closer the association between small molecule drugs and target genes. Small molecule drugs with *p* < 0.05 and higher comprehensive scores are considered significant (20).

### 2.9 Molecular docking

Molecular docking verification: 2D structures of the core components, Boldine and piceatannol, were obtained from the PubChem database. NAE1 (1TT5) protein conformational screening was performed in the RCSB PDB database^3^, and 3D structure in PDB format were downloaded. These structures were imported into PyMol software for water and small molecule ligand removal, and hydrogen addition, charge addition, and nonpolar hydrogen synthesis were performed in Autodock Tools software. Finally, molecular docking was performed.

## 3 Results

### 3.1 General information on participants

As shown in Table 1, a total of 2,234 participants were included in the study, including 1,160 in the control group and 1,074 in the MCI group. Statistical analysis of the two groups revealed that age, gender, smoking, education level, monthly household income, living arrangements, mobile phone use, pet ownership, and stress may be factors influencing MCI (*p* < 0.05).

**Table 1:** General information of the respondents.

Variable	CTL (*n* = 1,160)	MCI (*n* = 1,074)	t/*χ2* /*Z*	*p*
Age	68.45 ± 7.06	69.94 ± 6.67	−5.108	**< 0.001**
Gender				
Male	470 (40.52%)	385 (35.85%)	5.230	**0.022**
Female	689 (59.40%)	689 (64.15%)		
Smoking				
Yes	623 (53.71%)	294 (27.37%)	76.128	**< 0.001**
No	450 (38.79%)	740 (68.90%)		
Drinking				
Yes	168 (14.48%)	57 (5.31%)	52.268	**< 0.001**
No	984 (84.83%)	427 (39.76%)		
Educational level				
NFE	33 (2.84%)	21 (1.96%)	1.169	0.280
NGFES	54 (4.66%)	24 (2.23%)		
GFES	164 (14.14%)	104 (9.68%)		
GFJHS	500 (43.10%)	573 (53.35%)		
GTT	288 (24.83%)	281 (26.16%)		
GFJC	84 (7.24%)	44 (4.10%)		
GFBD	36 (3.10%)	26 (2.42%)		
GDOA	1 (0.09%)	1 (0.09%)		
Marital status				
Married	987 (81.8%)	902 (83.99%)	0.066	0.798
Divorced	30 (3.6%)	38 (3.54%)		
Widowed	114 (12.1%)	112 (10.43%)		
Single	27 (1.9%)	20 (1.86%)		
Other	2 (0.6%)	2 (0.19%)		
Income				
≤ 5,000	289 (24.91%)	262 (24.39%)	3.783	0.052
5,000–10,000	660 (56.90%)	527 (49.07%)		
10,000–15,000	137 (11.81%)	214 (19.93%)		
15,000–20,000	20 (1.72%)	37 (3.45%)		
≥ 20,000	54 (4.66%)	34 (3.17%)		
Living style				
LA	536 (46.21%)	211 (19.65%)	436.508	**< 0.001**
LWS	433 (37.33%)	241 (22.44%)		
LWCAG	189 (16.29%)	617 (57.45%)		
LWCO	1 (0.09%)	0		
LWGO	1 (0.09%)	5 (0.47%)		
Other	0	0		
Mobile phone usage	26.26 ± 10.32	21.52 ± 9.01	11.572	**< 0.001**
Pet keeping	9.68 ± 20.81	8.06 ± 17.90	1.978	**0.048**
Stress	16.14 ± 4.22	15.69 ± 3.67	2.733	**0.006**
Anxiety	15.97 ± 3.66	16.81 ± 4.29	−4.989	**< 0.001**
Depression	15.46 ± 3.52	16.11 ± 3.79	−4.162	**< 0.001**
AD				
No dementia	1,160 (100%)	750 (69.83%)	496.364	**< 0.001**
Suspected dementia	0	322 (29.98%)		
Mild dementia	0	2 (0.19%)		

NFE, No formal education; NGFES, Not graduated from elementary school; GFES, Graduated from elementary school; GFJHS, Graduated from junior high school; GTT, Graduated from high school/technical secondary school/technical school; GFJC, Graduated from a junior college; GFBD, Graduated from a bachelor’s degree; GDOA, Graduate degree or above; LA, Living alone; LWS, Living with a spouse; LWCAG, Living with children and grandchildren; LWCO, Living with children only; LWGO, Living with grandchildren only. “Suspected dementia” refers to participants with mild cognitive impairment (MCI) whose clinical characteristics suggest prodromal symptoms of Alzheimer’s disease, but who meet the criteria for mild cognitive impairment (CDR = 0.5) rather than dementia (CDR≥1). The meaning of bold values is less than 0.05.

### 3.2 Correlation analysis of various factors

**Figure 1:**
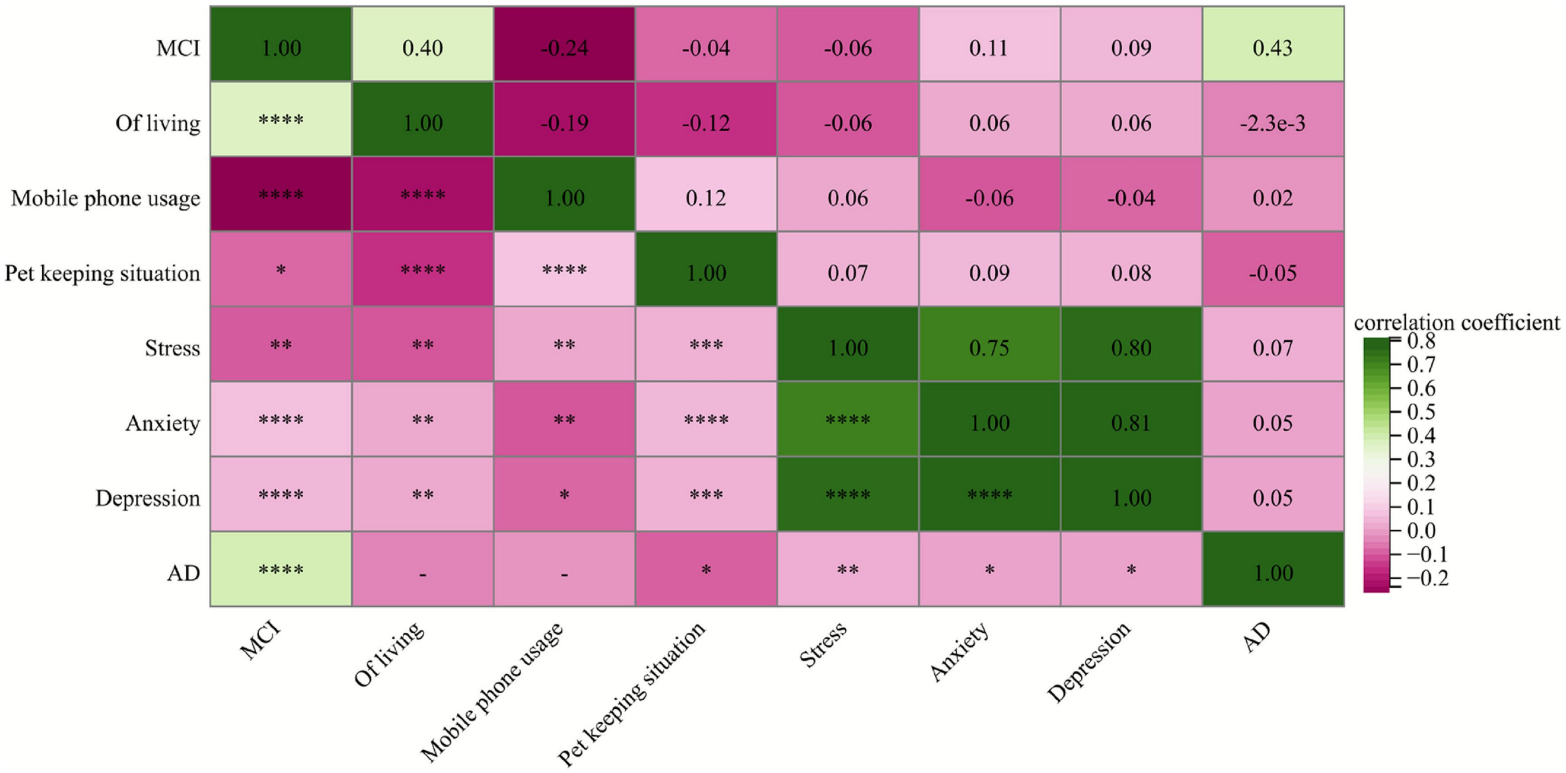
Correlation analysis of various factors. *, *p* < 0.05; **, *p* < 0.01; ***, *p* < 0.001.

Pearson correlation analysis showed that MCI was significantly positively correlated with living style, anxiety, depression, and AD (*p* < 0.05); and significantly negatively correlated with mobile phone use, pet ownership, and stress (*p* < 0.05). AD was significantly negatively correlated with pet ownership and significantly positively correlated with stress, anxiety, depression, and MCI (*p* < 0.05) (Figure 1).

### 3.3 WGCNA screening of MCI signature genes

**Figure 2:**
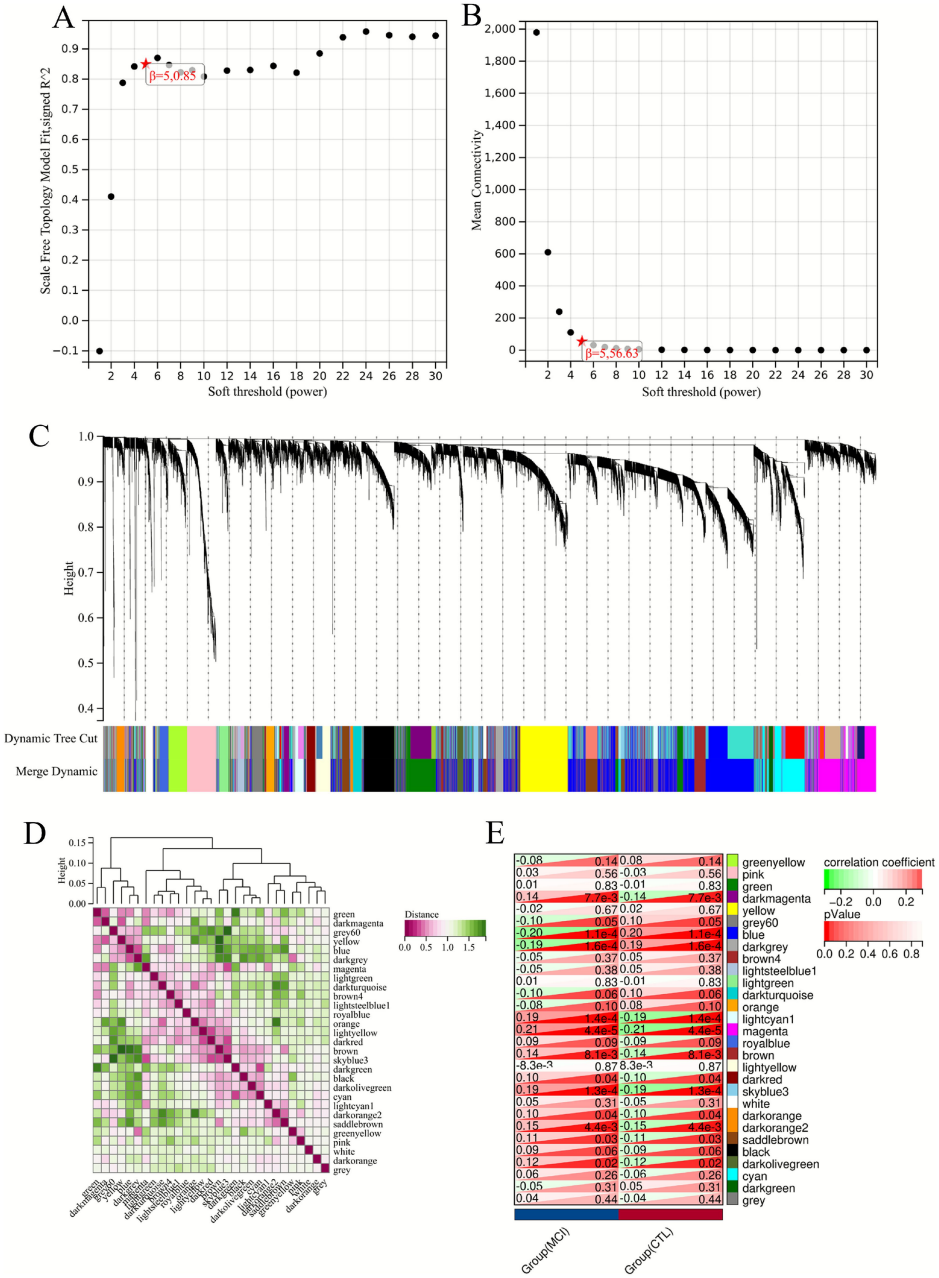
Co-expression analysis based on the WGCNA algorithm (GSE140829). **(A,B)** Determination of the soft threshold (*β*) to establish a scale-free topology and optimal connectivity. *β* was set to 5. **(C)** Hierarchical clustering dendrogram shows the classification of genes into 29 distinct modules. Each color below the dendrogram represents a module. **(D,E)** Heat map showing the relationship between module features and phenotypes.

To identify signature genes associated with MCI, we first performed WGCNA analysis using the GSE140829 dataset as the training set. After preliminary sample clustering, no outliers were detected (Figure 2A). Optimal scale-free connectivity was established by setting *β* = 5 (Figure 2B), and 29 gene modules were identified through hierarchical clustering (Figure 2C). Eleven modules were found to be associated with MCI, with the blue and magenta modules showing the highest correlation with MCI (*p* < 0.05) (Figures 2D,E).

**Figure 3:**
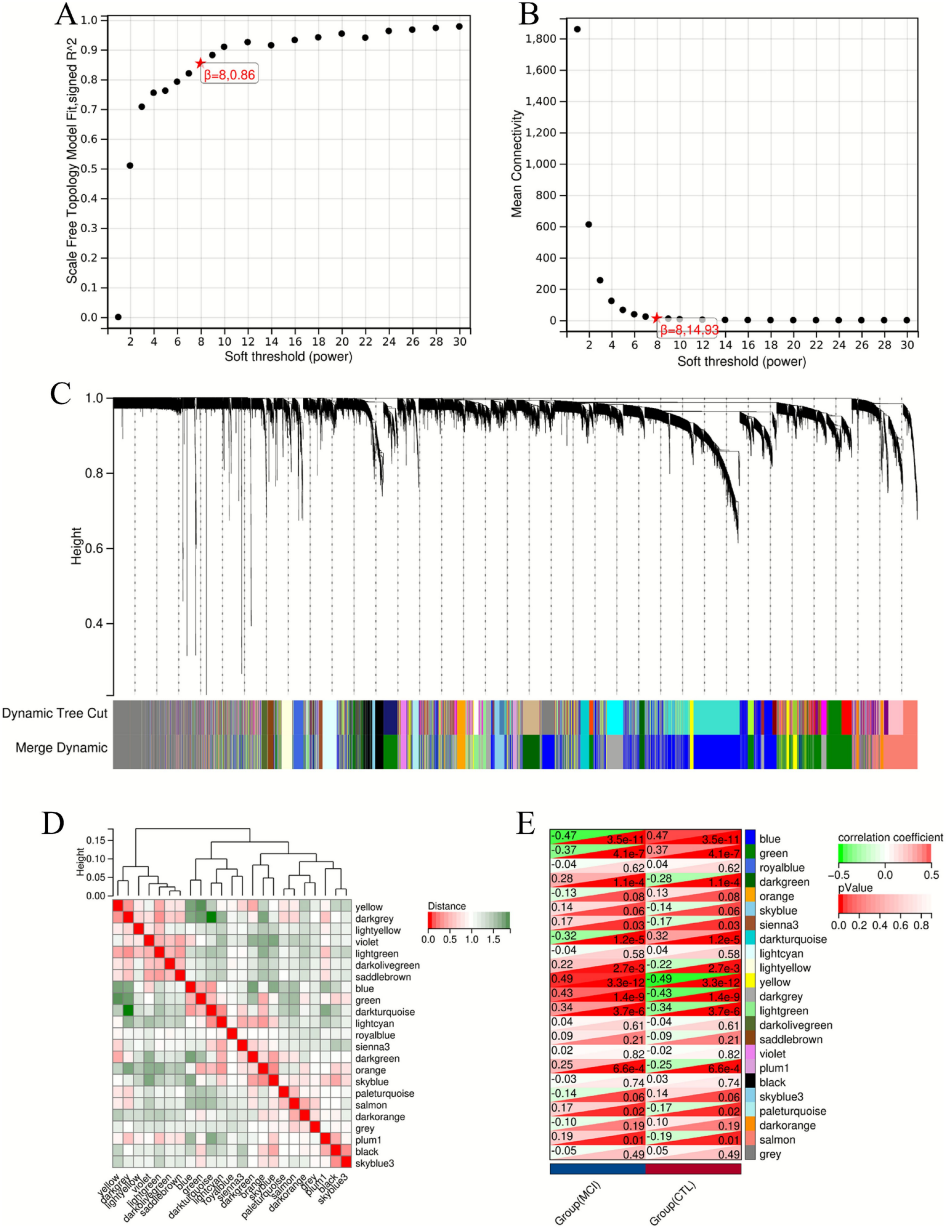
Co-expression analysis based on the WGCNA algorithm (GSE63060). **(A,B)** Determination of the soft threshold (*β*) to establish a scale-free topology and optimal connectivity. *β* was set to 8. **(C)** Hierarchical clustering dendrogram showed the classification of genes into 29 distinct modules. Each color below the dendrogram represented a module. **(D,E)** Heat map showed the relationship between module features and phenotypes.

**Figure 4:**
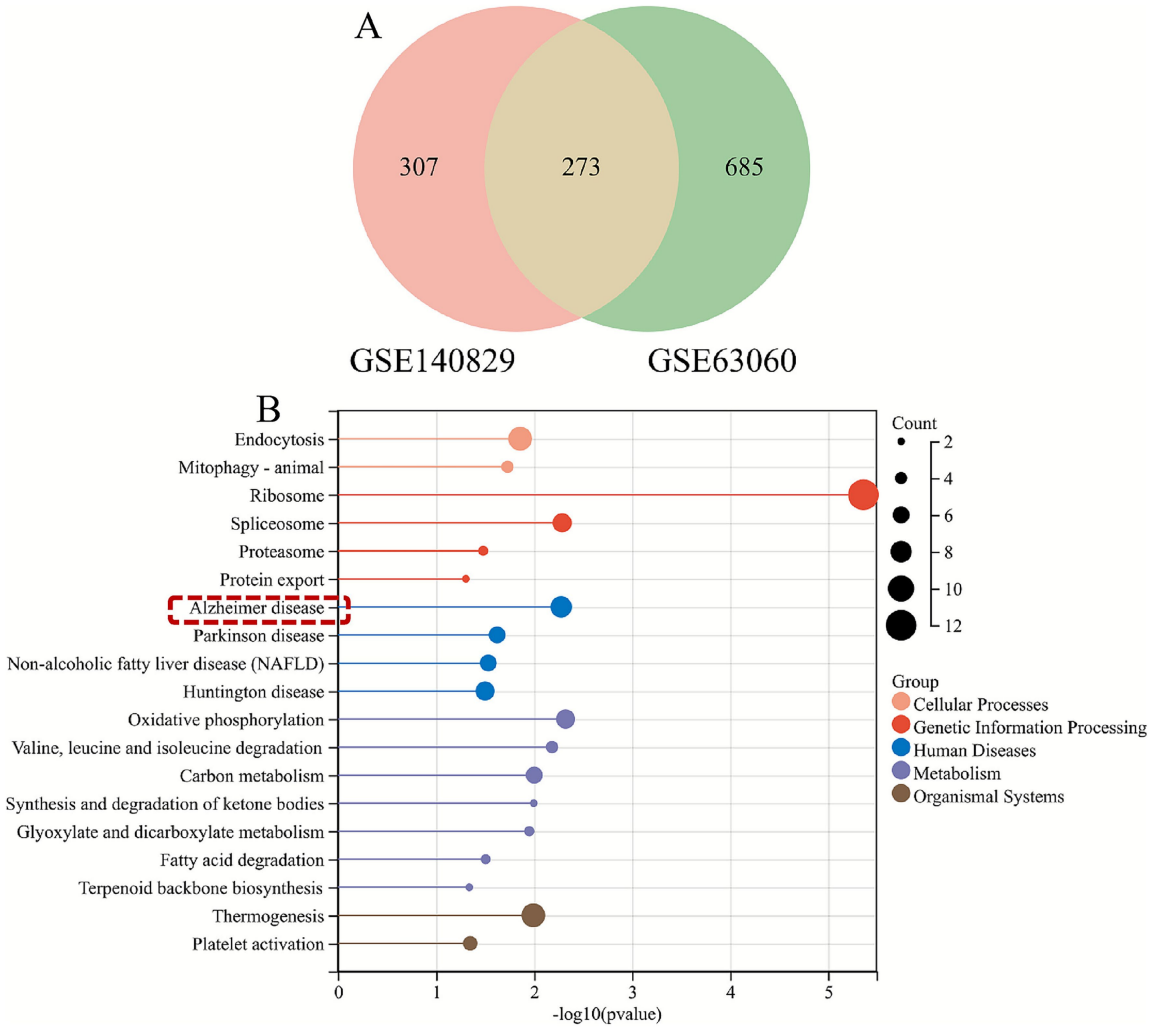
KEGG pathway analysis of MCI characteristic genes. **(A)** VENN graph showing the hub genes intersection between datasets. **(B)** KEGG pathway analysis of MCI characteristic genes.

Genes within these modules were aggregated to yield 580 hub genes. WGCNA analysis was then performed using the GSE63060 dataset as the validation set. Optimal scale-free connectivity was established by setting *β* = 8 (Figures 3A,B), and 23 gene modules were identified through hierarchical clustering (Figure 3C). A total of 12 modules were associated with MCI, with the blue and yellow modules showing the highest correlation with MCI (*p* < 0.05) (Figures 3D,E). The hub genes in the module were cross-validated with the 580 hub genes in the dataset GSE140829, and a total of 273 hub genes were obtained (Figure 4A).

### 3.4 Co-expression analysis of KEGG and Alzheimer’s disease pathway genes in MCI signature genes

**Figure 5:**
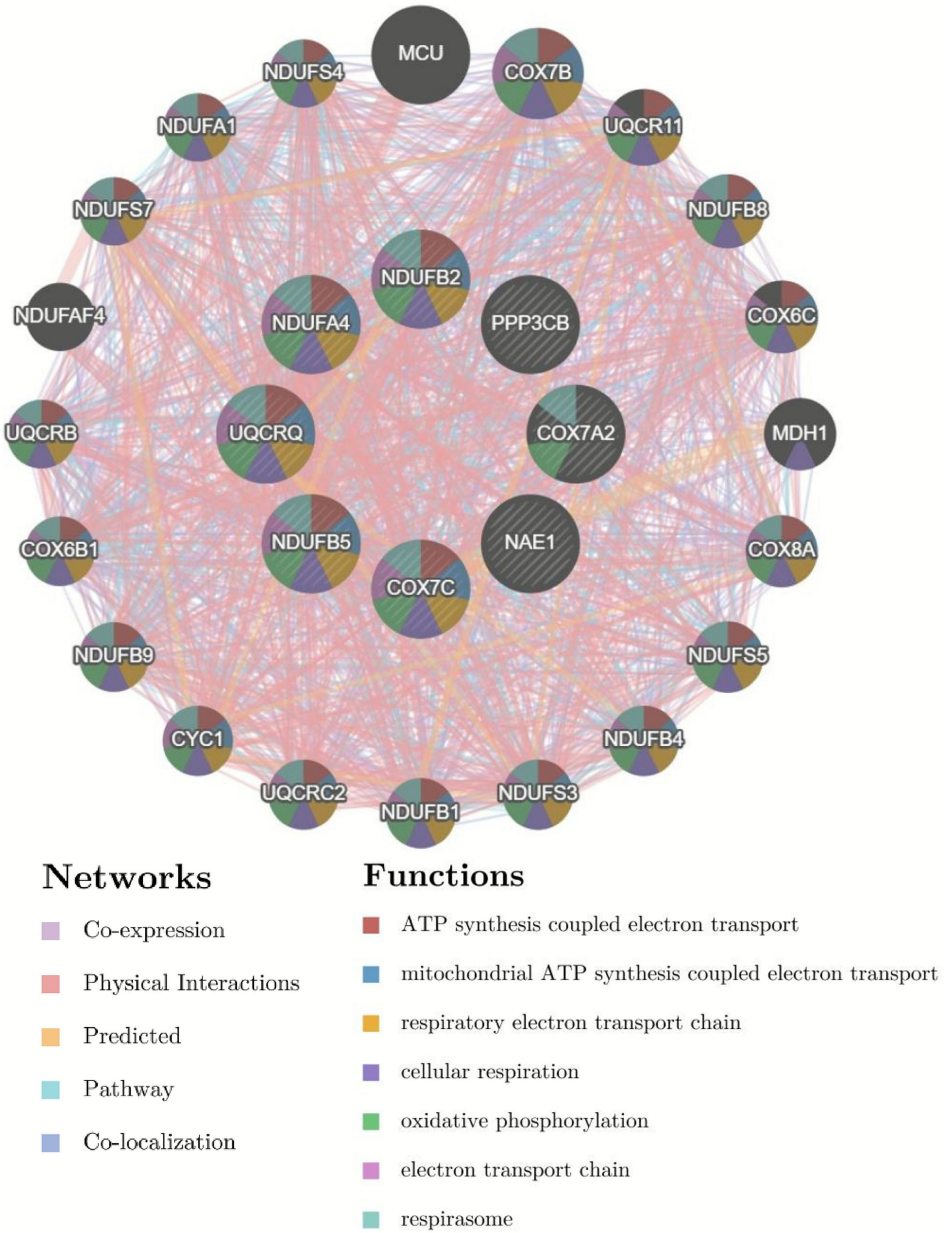
GeneMANIA analysis of relevant interactive genes of feature gene.

KEGG pathway analysis of MCI signature genes revealed enrichment in four diseases, including Alzheimer’s disease, within the Human Diseases category, three of which are neurodegenerative diseases. Alzheimer’s disease ranked first within the Human Diseases category, suggesting possible comorbidity between MCI and Alzheimer’s disease (Figure 4B). Therefore, co-expression analysis of Alzheimer’s disease pathway genes was further performed. As shown in Figure 5, eight Alzheimer’s disease pathway genes (COX7A2, COX7C, NAE1, NDUFA4, NDUFB2, NDUFB5, PPP3CB and UQCRQ) interacted with 20 genes.

### 3.5 Screening for pathway gene targeted interventions

As shown in Table 2, we list the top 10 targeted interventions identified based on pathway gene screening. Boldine and piceatannol were both natural compounds, and interestingly, they share a common target gene NAE1. Given the advantages of natural compounds such as low toxicity and minimal side effects, these two interventions may be potential targeted drug candidates for the treatment of MCI.

**Table 2:** Top 10 targeted interventions.

Term	*p*	Adjusted *p*	Odds ratio	Combined score	Target gene
Hydralazine	0.003	0.032	14.92	87.61	UQCRQ; NDUFB2; COX7C
Ampyrone	0.003	0.032	14.82	86.75	UQCRQ; NDUFB2; COX7C
Boldine	0.006	0.063	190.26	961.58	NAE1
Griseofulvin	0.009	0.079	135.86	643.51	NAE1
5-Amino-2-naphthalenesulfon-ic acid	0.010	0.079	124.03	576.75	NAE1
1,9-Pyrazoloanthrone	0.011	0.085	105.63	475.00	NAE1
Amlodipine	0.012	0.086	95.06	417.81	NAE1
Piceatannol	0.014	0.086	83.86	358.46	NAE1
Theophylline	0.014	0.086	13.85	58.94	PPP3CB; NDUFB2
Benzylpenicillin	0.015	0.086	75.02	312.60	PPP3CB

### 3.6 Molecular docking

**Figure 6:**
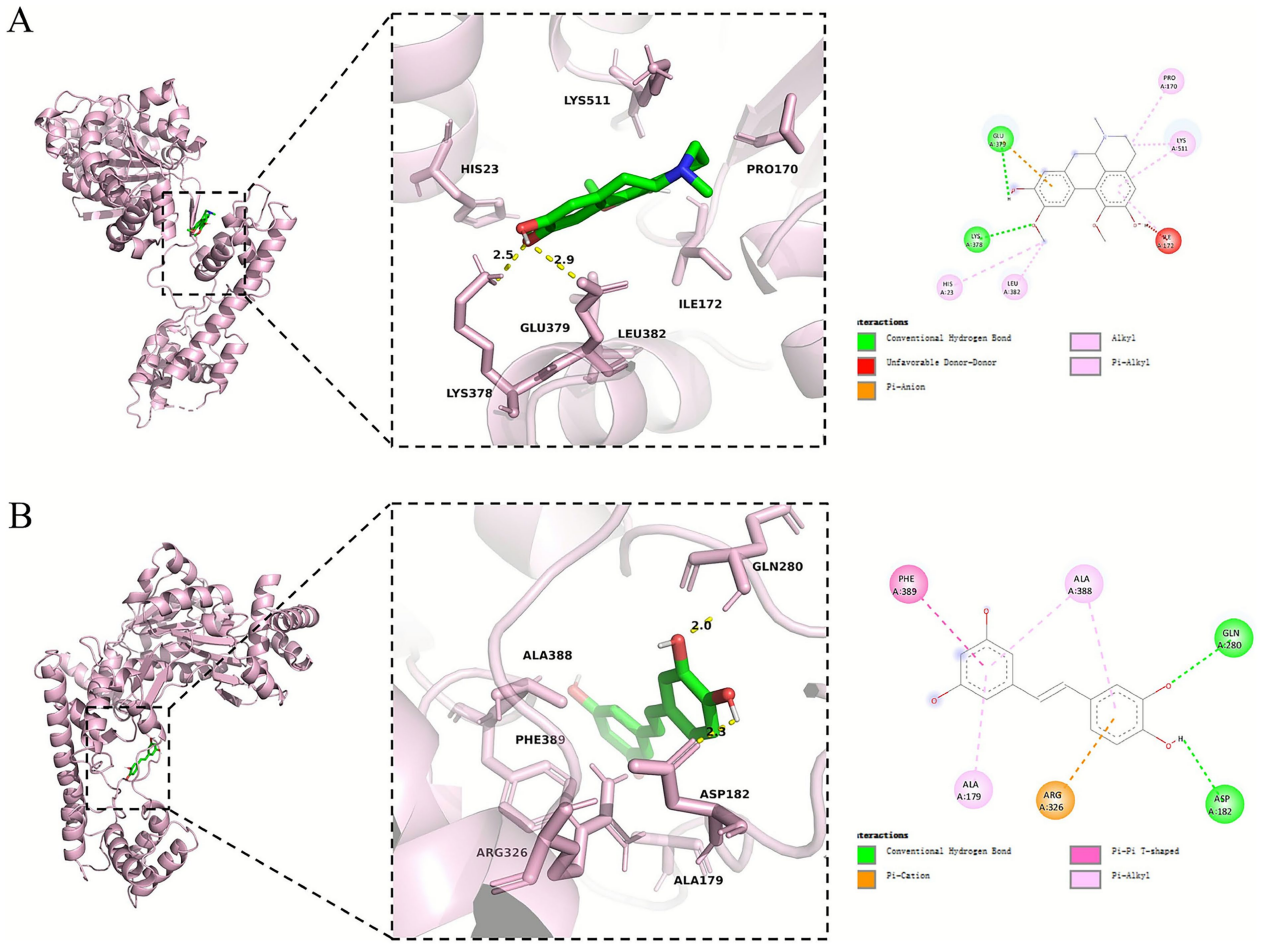
Molecular docking model of Boldine and Piceatannol with NAE1. **(A)** Boldine-NAE1. **(B)** Piceatannol-NAE1.

As mentioned above, the targeted intervention agents Boldine and piceatannol share a common target gene, NAE1. Further, we performed molecular docking studies of Boldine-NAE1 and piceatannol-NAE1 to evaluate their binding capabilities. The binding energies of boldine, piceatannol, and NAE1 were −7.429 and −7.157 kcal/mol, respectively, both below −5 kcal/mol. The negative binding free energy indicated that the ligand and acceptor could bind spontaneously, and the larger absolute value corresponded to higher stability of the complex (Figure 6). These results suggested that boldine, piceatannol, and NAE1 all exhibited strong binding activity.

## 4 Discussion

Based on survey data from 2,234 community-dwelling elderly individuals, this study conducted analysis and intervention of comorbid characteristics between MCI and AD in a community-based population in Pudong New District, Shanghai for the first time. The results showed a significantly higher prevalence of AD in the MCI group compared to the control group, as well as a significant positive correlation between MCI and AD. This finding validates the clinical significance of MCI as a pre-stage of AD. MCI patients in the community were at high risk for developing AD and warrant priority intervention. This study found that age, gender, smoking, living style, mobile phone use, pet keeping, stress, anxiety, and depression are key correlates of MCI. Many of the results were consistent with existing studies, but also have community specificity: ① Age was a classic risk factor for cognitive impairment. The average age of the MCI group in this study was significantly higher than that of the control group, which is consistent with the conclusion of the Global Study on Aging and Cognitive Impairment that “the risk of MCI increases by approximately 30% with every 5-year increase in age” (21); ② The prevalence of MCI in women was higher than that in men, which may be related to the reduction of neuroprotective effects caused by the decline in estrogen levels in women after menopause (22, 23). However, community women were more inclined to “live with their children and grandchildren.” Whether this living style can mitigate cognitive decline through social support still needs further analysis; ③ Mobile phone use and pet keeping were associated with a reduced risk of MCI. This may be because mobile phone use helps maintain cognitive flexibility, while pet keeping can reduce loneliness through emotional companionship. Both factors protect cognitive function via the “cognitive stimulation-psychological regulation” pathway (24, 25). This suggests a low-cost and easy-to-promote strategy for community community-based intervention.

It is worth noting that living style is one of the most strongly correlated factors in this study: the proportion of “living with children and grandchildren” in the MCI group (57.45%) was significantly higher than that in the control group (16.29%), while the proportion of those “living alone” (19.65%) was significantly lower than that in the control group (46.21%), which is different from the traditional conclusion that “living alone increases the risk of cognitive impairment” (26). The reason for this may be that the elderly who “live with children and grandchildren” in the community of Pudong New Area in Shanghai were more likely to bear the responsibility of “taking care of grandchildren.” The physical and psychological burden caused by long-term high-intensity care may offset the protective effects of family support. This “care stress-cognitive decline” association has also been reported in previous community studies (27). Notably, although the MCI group was more likely to live with and care for grandchildren-a role associated with substantial objective burden-their self-reported stress levels were significantly lower than those of controls. This paradox may reflect diminished stress awareness or reporting bias in individuals with MCI, consistent with prior evidence of impaired emotional self-monitoring in early cognitive decline (28). While traditional models emphasize loneliness or social isolation as risk factors, our data suggest that high-contact but high-burden family roles may also confer risk, particularly when subjective distress is masked by cultural norms or neurocognitive changes. Future interventions should therefore consider not only socially isolated elders but also ‘hidden’ caregivers whose objective strain is not captured by standard stress questionnaires.

To analyze the molecular basis of the comorbidity between MCI and AD, this study identified 273 MCI characteristic genes through WGCNA analysis, among which the blue module of the training set and the blue/yellow module of the validation set were the core modules most related to MCI. This result reflects the reliability of the study. Different data sets can still identify consistent core gene modules across different soft thresholds, suggesting that these genes may be “conservative molecular markers” for the occurrence and progression of MCI. KEGG pathway analysis further revealed the key mechanism of the comorbidity between MCI and AD: MCI characteristic genes were significantly enriched in neurodegenerative disease pathways (including AD pathways), and the AD pathway ranking first in the “Human Diseases” category. This finding confirmed the comorbid nature of MCI and AD at the molecular level. AD-related molecular pathway disorders already exist in the MCI stage, such as *β*-amyloid protein (Aβ) deposition and abnormal signaling pathways associated with tau protein hyperphosphorylation (29). The results of the targeted intervention screening based on AD pathway genes identified Boldine and piceatannol were the top 10 candidate intervention agents, and both were natural compounds, both of which are natural compounds and have strong binding ability to NAE1. Boldine is a benzylisoquinoline alkaloid extracted from the plant of the genus Piper, which has demonstrated antioxidant and anti-inflammatory effects (30–32). *In vivo* animal experiments showed that Boldine can directly interact with Aβ, preventing the endoplasmic reticulum and mitochondria from being disturbed due to elevated Ca^2+^, thereby improving mitochondrial function, prevents synaptic failure and organelle dysfunction, and exerting a neuroprotective effect (33). Interestingly, taking Boldine for 7 consecutive days can significantly improve the learning and memory abilities of young and old mice, which may be achieved by inhibiting brain acetylcholinesterase activity and alleviating brain oxidative stress (34). Long-term oral administration of Boldine to AD in model mice can significantly inhibit the abnormal upregulation of glial cell hemichannel activity during the pathological process, downregulate the excessive activation of Ca^2+^ signals in astrocytes, and reduce the abnormal release of extracellular adenosine triphosphate (ATP) and the excitatory neurotransmitter glutamate, effectively alleviating the pathological damage of neurons in the hippocampus of AD mice (35).

Piceatannol is a resveratrol analogue that can improve cognitive function in AD model mice by inhibiting tau protein aggregation (36). In the Aβ-induced PC12 cell injury model, piceatannol significantly reduced the abnormal accumulation of intracellular ROS and inhibited Aβ-mediated cell apoptosis characteristics (such as internucleosomal DNA fragmentation, nuclear condensation, PARP cleavage and caspase-3 activation). The results suggest that piceatannol can protect PC12 cells by blocking Aβ-induced ROS generation and alleviating oxidative stress (37). In the Aβ-induced PC12 cell apoptosis model, piceatannol can activate the PI3K/Akt/Bad signaling pathway and regulates its downstream mitochondria-mediated caspase-dependent apoptosis pathway, ultimately exerting a protective effect against Aβ-induced PC12 cell apoptosis (38). In addition, piceatannol can also protect HT22 neuronal cells from glutamate-induced cell death, at least in part, by inducing Nrf2-dependent HO-1 expression (39). In this study, the combined efficacy scores of the two drugs were significantly higher than those of other synthetic drugs. Given their low toxicity, they are more suitable for long-term intervention in MCI patients. In addition, boldine and piceatannol can simultaneously target multiple pathways of “oxidative stress-inflammation-protein aggregation” (30, 36). The pathogenesis of AD has multi-pathway synergistic pathways. Single-target drugs are difficult to effectively block disease progression. In contrast, the multi-target advantages of natural compounds can enhance intervention effect. Some foods rich in these compounds (such as grapes and peppers) can be used as carriers of community nutritional interventions. When combined with previously discovered lifestyle interventions such as mobile phone use and pet keeping, a nutrition-lifestyle-drug trinity community cognitive impairment prevention system is formed.

While this study has made progress in integrating community population data and transcriptomics analyses, it still has the following limitations. 1. The study participants were only from 14 communities in Pudong New District, Shanghai. This geographical limitation may make it difficult to generalize the results to other regions (such as rural areas or communities in the central and western regions). Future multicenter, cross-regional community cohort studies are needed. 2. This study was cross-sectional in design and could not determine the causal relationships between “influencing factors (such as living style and stress) and MCI” or “characteristic genes and comorbidities.” Further verification is needed through prospective cohort and molecular experiments. 3. This study only utilizes a self-rated stress scale, without incorporating objective caregiving time or physiological stress indicators (such as cortisol). Future research could assess stress from multiple dimensions. 4. The transcriptome data in the GEO database were derived from peripheral blood, not brain tissue. Differences in gene expression between peripheral samples and the central nervous system may have led to some key pathways not being detected. 5. The “characteristic genes” were derived from public databases, not from clinical blood samples of the surveyed population. In the next step, we will collect clinical blood samples from the surveyed population to enhance the relevance of the research, and this work has already passed ethical review.

## 5 Conclusion

Community-based MCI patients are at high risk for AD, and their comorbidity profile is influenced by multidimensional lifestyle and psychological factors. Boldine and piceatannol may be potential natural compounds for interventions in cognitive impairment. These findings provided a theoretical basis for early prevention and targeted intervention of cognitive impairment in the community.

## Data Availability

The datasets presented in this study can be found in online repositories. The names of the repository/repositories and accession number(s) can be found in the article/supplementary material.

## Footnotes

1
https://www.ncbi.nlm.nih.gov/geo/

2
http://genemania.org

3
https://www.rcsb.org/
